# Temporal Predictions from Anhedonia To Anxiety in Adolescents with Major Depressive Disorder

**DOI:** 10.1007/s10802-025-01362-6

**Published:** 2025-08-27

**Authors:** Yannie D. Lee, Kenneth Towbin, Daniel S. Pine, Argyris Stringaris, Katharina Kircanski

**Affiliations:** 1 Emotion and Development Branch, National Institute of Mental Health, Bethesda, MD USA; 2 University College London, London, UK; 3 Emotion and Development Branch, National Institute of Mental Health, Bethesda, MD USA

**Keywords:** Anhedonia, Adolescence, Depression, Anxiety, Comorbidity

## Abstract

Anhedonia is a hallmark symptom of major depressive disorder (MDD) characterized by diminished ability to experience pleasure, motivation, or interest in usual activities. Anhedonia also is recognized as a transdiagnostic symptom dimension, but its links to other symptom dimensions are not fully elucidated. Given the high rates of depression and anxiety comorbidity in adolescence, we assessed concurrent and longitudinal associations between anhedonia and anxiety symptoms in adolescents diagnosed with MDD. The sample consisted of 157 adolescents with MDD (*M*_age_ = 15.54, 71.34% female) who were followed for several years in a research context involving longitudinal observation and, for a portion of the sample, treatment. Participants regularly completed self-report measures of anhedonia and anxiety symptoms across time. Data were analyzed using multilevel modeling to examine within-person concurrent and temporally lagged effects. Findings indicated that anhedonia was concurrently associated with both social anxiety and generalized anxiety symptoms. Additionally, anhedonia predicted *only* social anxiety symptoms over time—such that anhedonia at the previous time point predicted subsequent social anxiety but not generalized anxiety. However, neither social anxiety nor generalized anxiety predicted anhedonia temporally. These results suggest that anhedonia may be one driver of comorbid *social* anxiety symptoms in adolescents with MDD. Putative mechanistic links warrant further investigation, including during transdiagnostic treatments for emotional disorders in adolescents. We highlight avenues for future research and intervention in adolescent MDD.

**Supplementary Information:**

The online version contains supplementary material available at 10.1007/s10802-025-01362-6.

## Introduction

Anhedonia is a core symptom of major depressive disorder (MDD) that manifests as a pervasive reduction in the ability to derive pleasure, motivation, or interest from usual activities (American Psychiatric Association [APA], 2022). In adult samples, anhedonia has emerged as a transdiagnostic symptom dimension, implicated across a wide range of psychopathology, such as attention-deficit hyperactivity disorder, autism spectrum disorder, schizophrenia, and substance use disorders (Guineau et al., 2023); Liang et al., 2022). However, its role in MDD is particularly pronounced, as anhedonia is a predictor of poor psychosocial outcomes (Wong et al., 2024), psychological and pharmacological treatment response (McMakin et al., 2012); Spijker et al., 2001); Treadway & Zald, 2011); Vrieze et al., 2013), and heightened risk for suicide-related outcomes (Auerbach et al., 2022); Nock & Kazdin, 2002); Winer et al., 2014). Despite its clinical significance, anhedonia remains largely unaddressed by existing cognitive and behavioral therapies (Cuijpers et al., 2014); Pizzagalli, 2022); Sandman & Craske, (Sandman and Craske 2022), which primarily target reductions in negative affect rather than enhancements in positive affect or hedonic capacity. While anhedonia has been extensively studied in adults, far less is known about its role in youth, particularly in relation to anxiety and depression comorbidity. This gap is especially relevant for adolescents with MDD, a population in which anhedonia may play a central role in the etiology and maintenance of depression, and may also contribute to the frequent comorbidity between depression and anxiety (Gupta et al.,(Gupta et al. 2024a). Given anhedonia’s potential role in exacerbating both depression and anxiety, the current study examined its relationship with anxiety in adolescents with MDD, conceptualizing it as a key mechanism underlying their comorbidity.

Depression and anxiety are highly comorbid in youth (Kalin, 2021); Melton et al., 2016). Studies indicate that approximately 25–50% of youth with depression also meet criteria for an anxiety disorder (Garber & Weersing,(Garber and Weersing 2010), while about 10–15% of those with an anxiety disorder experience comorbid depression (Cummings et al., 2014), underscoring the frequent co-occurrence and clinical significance of these conditions in early development (Long et al., 2018).

Despite the high prevalence and clinical impact of depression-anxiety comorbidity in youth, research has largely overlooked the mechanistic role of anhedonia in this relationship. Studies have established that comorbid anxiety amplifies the severity and chronicity of depression (Aderka et al., 2012); Franco et al., 2007); Koyuncu et al., 2019); O’Neil et al., 2010), and risk for various adverse outcomes such as suicidality (Nock et al., 2010), yet few have examined how anhedonia contributes to this exacerbation in adolescent populations. Given anhedonia’s association with poor treatment response (McMakin et al.,(McMakin et al. 2012) and heightened clinical severity in MDD (Gabbay et al., 2015), it is plausible that it also plays a central role in sustaining or worsening comorbid anxiety, further complicating clinical outcomes. These effects may be particularly pronounced during adolescence, a developmental stage marked by heightened vulnerability to mood and anxiety disorders.

Adolescence is a period of heightened vulnerability to anxiety and depression (Garber & Weersing, 2010); Johnson et al., 2018); McLaughlin & King, 2015). This coincides with enhanced plasticity in reward-responsive brain areas (Foulkes & Blakemore, 2016); Galván, 2010, 2013); Walker et al., 2017), which may increase sensitivity to disruptions in hedonic processes. Given the interplay between neurodevelopment and psychopathology, adolescence may be a particularly sensitive period for the emergence and trait stabilization of anhedonia, influencing both depression and anxiety trajectories. For example, one study found that adolescent anhedonia was uniquely associated with more severe clinical outcomes, including greater overall illness severity, higher suicidality scores, longer episode duration, and a greater number of depressive episodes (Gabbay et al., 2015). A clinical trial examining treatment-resistant adolescents revealed that anhedonia uniquely predicted a longer time to remission and fewer depression-free days during second-step treatment with selective serotonin reuptake inhibitors (SSRIs) and cognitive-behavioral therapy (McMakin et al., 2012). Furthermore, preliminary findings suggest that anhedonia may follow distinct developmental trajectories across adolescence, with some adolescents experiencing stable or decreasing symptoms while others exhibit persistence or worsening over time (Gupta et al., 2024b). Among at-risk adolescents, lower depression severity emerged as a potential protective factor, buffering against the persistence or exacerbation of anhedonia as they transitioned into adulthood. These variations in anhedonia trajectories suggest that some adolescents may be more resilient to its long-term effects, while others remain vulnerable to worsening symptoms that contribute to later psychopathology. Altogether, these findings underscore the clinical significance of anhedonia in adolescent MDD and impact on illness severity, clinical trajectories, and treatment outcomes.

Historically, theoretical models of anxiety and depression have characterized anhedonia as a facet of low positive affect, a trait unique to depression (e.g., Clark & Watson, 1991). However, reconsidering anhedonia within a transdiagnostic framework may provide new insights into the mechanisms underlying co-occurring depression-anxiety in adolescence. For instance, Winer and colleagues (2017) conducted three studies examining the interplay among anhedonia, anxiety, and depressive symptoms in a non-clinical sample of adults. They found that anxiety-driven avoidance moderated the relationship between anxiety and depression, particularly among individuals who avoided highly enjoyable activities, ultimately leading to increased depressive symptoms. Furthermore, they demonstrated that anxiety mediated both cross-sectional and longitudinal associations between anhedonia and depression, with this mediation emerging consistently across multiple timepoints. Notably, anxiety symptoms emerged as a key mediator linking recent increases in anhedonia to later depressive symptoms. This highlights the central role of anxiety in the pathway from reward dysfunction to depression and suggests it may be a mechanism through which changes in hedonic capacity shape depressive symptom trajectories.

In a complementary vein, recent work has identified how anhedonia may also impede recovery from anxiety by disrupting reward-related processes essential to therapeutic success. Specifically, anhedonia has been proposed as a barrier to recovery in anxiety due to its influence on reward valuation, responsiveness, and learning. For example, diminished reward valuation may lead individuals to perceive potential rewards as less attainable or worthwhile, tipping the balance in favor of avoidance over approach behaviors (Pittig et al., 2021). Moreover, reduced responsiveness to rewards—either anticipated or experienced—may interfere therapeutic exposures to threat-relevant contexts, limiting opportunities for positive reinforcement and new learning (Rosenberg & Horowitz et al., 2024). As such, altered reward processing may represent a key mechanism through which anhedonia perpetuates both anxiety and depression, underscoring the need for further research into how specific anxiety symptoms are associated with hedonic deficits during critical periods of development.

Mixed findings link anhedonia to generalized anxiety disorder (GAD; Brown et al., 1998); Prenoveau et al., 2010), whereas stronger evidence supports its association with social anxiety disorder (SAD). SAD shares substantial clinical overlap with depressive symptomatology—including sadness, loneliness, poor self-esteem, and low positive affect—and is highly prevalent among youth with comorbid MDD (Adams et al., 2016; Danneel et al., 2019; Stein et al. 2005). Adults with severe social anxiety symptoms exhibit blunted reward responsiveness similar to that seen in depression (Kashdan, 2004, 2007) and report lower levels of both state and trait positive affect (Kashdan & Steger, 2006). Emerging evidence indicates that these associations are also present in adolescents (Bakker et al., 2017); Forbes & Dahl, 2012); Leventhal et al., 2017), suggesting that hedonic deficits may be rooted early on in development. Luckhardt and colleagues (Luckhardt et al. 2023) found that adolescents with comorbid MDD and SAD exhibited blunted reward anticipation and processing on a gambling task, mirroring patterns seen in adults with depression (Luking et al., 2016). Similarly, reward-related deficits in both social and non-social contexts have been linked to treatment response in youth with anxiety and depression, suggesting that altered reward processing may contribute to the persistence of anxiety symptoms over time (Schwartz et al., 2019). Despite these findings, research on anhedonia in adolescents with co-occurring MDD and anxiety remains scarce. Most existing studies have focused on adult, non-clinical samples and cross-sectional designs, limiting insight into how anhedonia relates to social and generalized anxiety over the course of development.

The present study aims to bridge this gap by examining the longitudinal relationships between anhedonia, social anxiety, and generalized anxiety in adolescents diagnosed with MDD. Specifically, we assess both concurrent and longitudinal relationships between anhedonia and anxiety symptoms. Anhedonia may maintain and exacerbate generalized anxiety by diminishing reward valuation and responsiveness, reinforcing avoidance behaviors, and perpetuating a maladaptive feedback loop (Taylor et al., 2022). As reward motivation diminishes, individuals may increasingly turn to avoidance as a coping mechanism. Although this may offer short-term relief, it can exacerbate anxiety and reduce engagement with potentially rewarding experiences over time (Rosenberg & Horowitz et al., 2024). Consequently, this pattern may hinder exposure to corrective experiences and perpetuate motivational and hedonic deficits. Social anxiety, a well-established risk factor for depression (Adams et al., 2016); Dalrymple & Zimmerman, 2007); Griffith et al., 2020); Kalin et al., 2021; Koyuncu et al., 2019); Ohayon & Schatzberg, 2010); Long et al., 2018); Rapee et al., 2019), may also arise from depression-related interpersonal challenges, such as peer rejection, social withdrawal, and impaired social skills (Cummings et al., 2014); Danneel et al., 2019); Hamilton et al., 2016); Jacobson & Newman, 2017). Consistent with cumulative interpersonal risk models of social anxiety and depression (Epkins & Heckler, 2011), repeated negative social experiences—such as low peer acceptance, social exclusion, or difficulty forming or maintaining relationships—may reinforce avoidance behaviors and further entrench comorbid symptoms over time. These dynamics are particularly salient in the context of adolescence, a critical period when peer relationships significantly shape psychosocial well-being and identity (Corsano et al., 2006); Lamblin et al., 2017); Oberle et al., 2010); Roach, 2018). First, we hypothesized that anhedonia would be positively associated with social and generalized anxiety symptoms concurrently. Second, we hypothesized that anhedonia would share a positive, reciprocal relationship with both social and generalized anxiety over time.

## Methods

### Participants

Data for the present study was collected as part of the Characterization and Treatment of Depression (CAT-D) study at the National Institute of Mental Health (NIMH), a longitudinal cohort study focused on the characterization and treatment of depression in adolescents (Sadeghi et al., 2022; CAT-D data openly available at https://github.com/transatlantic-comppsych/CATD-study). The cohort encompassed both adolescents with depression and nonpatient controls, monitoring their development and progress over the span of several years. Recruitment began in December of 2018. Participants were recruited primarily from the Washington metropolitan region, identified through locally distributed postcards aimed at identifying adolescents exhibiting symptoms of MDD. Community practitioner referrals were also utilized for recruitment. All participants were assessed by trained clinicians who utilized the Kiddie Schedule for Affective Disorders and Schizophrenia - Present and Lifetime Version (KSADS-PL; Kaufman et al., 1997), a semi-structured diagnostic interview. Out of 170 adolescents with MDD initially enrolled in the cohort, 157 completed the required assessments for inclusion and provided permission for data sharing in this analysis. Of these 157 adolescents, 122 completed the KSADS in-person, while 35 completed the face-to-face clinical KSADS interview using telehealth platforms due to COVID-19-related restrictions. Inclusion criteria consisted of individuals between the ages of 11 and 17 at the time of enrollment and who had met criteria for a previous or ongoing diagnosis of MDD according to the Diagnostic and Statistical Manual of Mental Disorders, Fifth Edition (DSM-5; APA,^2013^). Exclusion criteria included: a diagnosis of schizophrenia, schizophreniform disorder, schizoaffective illness, bipolar disorder, severe autism spectrum disorder, anorexia nervosa, or other severe eating disorders; an intelligence quotient (IQ) of < 70 on the Wechsler Abbreviated Scale of Intelligence - Second Edition (WASI II; Wechsler, (Wechsler 2011); depressive symptoms due to effects of drugs of abuse or a neurological condition; a diagnosis of alcohol or other substance use disorders; current active suicidal ideation; repeated self-harm in the context of interpersonal conflict; and serious medical conditions such as epilepsy or heart disease. All participants provided written consent as part of standard enrollment procedure and received monetary compensation for research task and questionnaire completion. As part of the study, adolescents with current diagnosis of MDD were also offered treatment in the form of evidence-based cognitive behavioral therapy (CBT), primarily consisting of behavioral activation (BA).

### Procedures

The study comprised two primary parts: (1) Characterization and (2) Treatment. In the Characterization portion, all participants engaged in various behavioral tasks and underwent neuroimaging scans (i.e., magnetic resonance imaging and magnetoencephalography), and completed structured follow-up assessments at approximately 6-, 12-, 18-, and 24-months post-enrollment. Individuals with ongoing major depression expressing interest treatment were given evidence-based CBT sessions on a weekly basis, for a maximum of 13 weeks. The intervention was based on incorporating BA with components of MATCH-ADTC (Chorpita & Weisz, 2009) and was designed as a 13–14 session protocol with each session lasting approximately 1.5–2 h. Given this variability in engagement and the fact that most participants did not complete a full treatment dose (*M* = 7.11), we did not separate participants into distinct treatment and non-treatment groups in our analyses. At each visit, participants were asked to complete several self-report measures including measures of anhedonia and anxiety symptoms (details below). All research procedures were approved by the Institutional Review Board (IRB). Due to the COVID-19 pandemic, a substantial portion of the data were collected during a period that only allowed for virtual visits. Additionally, the number of total visits for each participant varied (range = 1–25, *M* = 8.22, *SD* = 5.44), and days between visits were not standardized (range = 6-1758, *M* = 465.24, *SD* = 399.75). Test-retest reliability and internal consistency were calculated using all available timepoints, regardless of the number of visits per participant or the time between them. We computed single-measure two-way random-effects ICCs [ICC(2,1)] with 95% confidence intervals to evaluate absolute agreement across all available timepoints (Koo & Li, 2016). Internal consistency was assessed using Cronbach’s alpha (α) and McDonald’s omega (ω).

### Measures

#### Anhedonia

The Snaith-Hamilton Pleasure Scale (SHAPS; Snaith et al., (Snaith et al. 1995) is a 14-item self-report measure of anticipated hedonic response, or lack thereof, to typically pleasurable experiences. These experiences span sensory, social, and intrapersonal domains (e.g., “I would be able to enjoy my favorite meal”, “I would enjoy being with my family or close friends”, “I would find pleasure in my hobbies and pastimes”). The SHAPS has demonstrated robust internal consistency in clinical samples (α = 0.82-0.91; Franken et al., 2007); Langvik & Austad, 2019; Nakonezny et al., 2015) and adequate reliability in adolescent populations (α = 0.87; Leventhal et al., 2015).

Items are rated on a 4-point Likert-type scale (0 = *strongly agree*; 3 = *strongly disagree*) and yield a severity score ranging from 0 to 42, with higher scores indicating greater levels of anhedonia. The present study utilized total scores (internal consistency in present sample: *α* = 0.93, *ω* = 0.94). Test-retest analyses demonstrated moderate reliability across time (ICC = 0.61).

#### Social and Generalized Anxiety

The self-report version of the Screen for Child Anxiety Related Disorders (SCARED; Birmaher et al., 1997) was used to assess social and generalized anxiety. The SCARED consists of 41 items that screen for the presence of five anxiety disorders over the past three months: generalized anxiety disorder, separation anxiety disorder, panic disorder, social anxiety disorder, and school avoidance. Participants responded to a series of statements indicating the degree to which the statements described them (e.g., “I am nervous”, “People tell me that I worry too much”, “It is hard for me to talk with people I don’t know well”) on a 3-point Likert scale (0 =*not true or hardly ever true*; 1 = *somewhat true or sometimes true*; 2 = *very true or very often true*). The measure yields a total score as well as five subscale scores. The total SCARED has demonstrated excellent internal consistency (α = 0.91), while the social and generalized anxiety subscales have shown good reliability, with α = 0.78 and α = 0.81, respectively (Runyon et al., 2018). Subscale scores were used in all analyses, demonstrating strong internal consistency in the present sample (social anxiety: α = 0.91, ω = 0.93; generalized anxiety: α = 0.92, ω = 0.94). Test-retest analyses demonstrated good reliability across both subscales (social anxiety: ICC = 0.73; generalized anxiety: ICC = 0.76).

### Statistical Analyses

Data were analyzed using R (version 4.4.2; RStudio Team, 2024) and HLM 8.0 (Raudenbush & Congdon, 2021). Internal consistency and reliability analyses, including baseline Pearson correlations, Cronbach’s alpha, McDonald’s omega, and intraclass correlation coefficients (ICCs) were calculated using the psych package (version 2.4.12; Revelle, 2024). Statistical assumptions were tested using the dplyr package (version 1.1.4; Wickham et al., 2023). Power analyses were conducted using the pwr package (version 1.3-0; Champely, 2020). Data visualization was modeled using the ggplot2 package (version 3.3.3; Wickham, 2016). Given the hierarchical structure of the data in which time points were nested within participants, we employed multilevel modeling (MLM). MLM enabled simultaneous estimation of within- and between-person effects, while accommodating varying time intervals between time points, and accommodating missing data through implementation of restricted maximum likelihood estimation (REML) (Snijders & Bosker, 2011). For our analyses, we combined all available time points per participant (i.e., Characterization and Treatment, when applicable). This approach maximized the number of data points, bolstering statistical power. To account for variability between time points in time-lagged MLMs, a time-point difference variable was calculated corresponding to the number of days between consecutive time points (days) in the study and included as a level-1 covariate. Random intercepts and slopes were specified in all models. Robust standard errors were used. Level-1 predictors were centered around the group (person) mean, such that coefficients represent the relation between the predictor and outcome within person. In all equations below, i denotes time points and j denotes participants. As multiple statistical models were conducted, each with multiple predictors, all results were false discovery rate (FDR)-corrected (Benjamini-Hochberg procedure) with the percentage of false positives set to*q* = 0.05. As such, results reported in the text and tables represent raw coefficients and standard errors from the MLMs along with FDR-corrected *p* values.

### Assumptions of Statistical Models

Following the recommendations of Snijders and Bosker (2011), we assumed normality of level-1 residuals and level-2 random coefficients, homogeneity of variance at level-1, and a constant covariance matrix for level-2 random coefficients.

Distribution of level-1 residuals and level-2 random coefficients were assessed using Shapiro-Wilk tests. Results indicated significant deviations from normality for all models at level-1 (W range: 0.960–0.986, *p* <.001). For level-2 random coefficients, results were mixed. Cross sectional and longitudinal models where anhedonia predicted anxiety symptoms deviated significantly from normality (W range: 0.933–0.979, *p*s < 0.001) while longitudinal models where anxiety symptoms predicted anhedonia met normality assumptions (W range: 0.983–0.984, *p*s > 0.071. Q-Q plots suggested that residuals were normally distributed at the center but showed deviations at the tails (eFigure 1 in the Supplement).

Homogeneity of variance was assessed using Levene’s test. All models were significant (*F*s = 1.30–2.71, *p*s < 0.05), indicating heteroscedasticity at the participant-level. To evaluate whether the assumption of a constant covariance matrix for level-2 random coefficients was met, likelihood ratio tests were conducted to compare models with random intercepts only to models with both random intercepts and slopes. For all models, the likelihood ratio tests were significant (*p*s < 0.001), indicating that including random slopes significantly improved model fit and that the effects of predictors varied meaningfully across participants, suggesting heterogeneity in the covariance matrix.

### Concurrent Associations

We first tested for concurrent associations of anhedonia with social anxiety and generalized anxiety within persons, within time points (Fox & Weisberg 2019).

**Level-1 Models** (time point level; separate equations used for social anxiety and generalized anxiety):

Anhedonia_ij_ = β_0j_ + β_1j_(Social Anxiety or Generalized Anxiety) + r_ij_.

**Level-2 Model** (participant level):

β_0j_ = γ_00_ + u_0j_.

β_1j_ = γ_*10*_ + u_1j_.

Here, Anhedonia_ij_ refers to the level of anhedonia for participant j at time point i, β_1j_ refers to the association between level of social anxiety or generalized anxiety and level of anhedonia for participant j, and r_ij_ is the within-person residual variance. γ_00_ refers to the mean level of anhedonia across participants, γ_10_ refers to the mean association between level of social anxiety or generalized anxiety and anhedonia across participants, and u_0j_ and u_0j_ are the respective between-person random effects.

### Temporal Associations

We next texted for time-lagged associations of anhedonia with social anxiety and generalized anxiety within persons. Sample equation:

**Level-1 Models** (time point level; four separate models conducted):

Anhedonia_ij(t)_ = β_0j_ + β_1j_(Social Anxiety_t−1_) + β_2j_(Anhedonia_t−1_) + β_3j_(Days Between t and t-1_t_) + r_ij_.

**Level-2 Models** (participant level):

β_0j_ = γ_00_ + u_0j_.

β_1j_ = γ_10_ + u_1j_.

β_2j_ = γ_20_ + u_2j_.

β_3j_ = γ_30_ + u_3j_.

Here, β_1j_ refers to the prediction of anhedonia at the current time point (t) by social anxiety at the previous time point (t-1). The analysis covaries β_2j_ as the level of anhedonia at the previous time point (t-1), such that results for β_1j_ reflect the effect of social anxiety on subsequent change in level of anhedonia. The analysis also covaries β_3j_, the number of days in between t and t-1.

### Power Analyses

We estimated power for key fixed effects in cross-sectional and longitudinal designs, assuming a one-sample t-test framework adjusted for clustering. Analyses targeted 80% power at α = 0.05 to detect small (*d* = 0.2), medium (*d* = 0.5), and large (*d* = 0.8) effect sizes (Cohen, 1988), with an assumed average of 7 observations per participant. Results indicated that detecting a small effect (*d* = 0.2) required 397 total observations (57 participants), a medium effect (*d* = 0.5) required 67 observations (10 participants), and a large effect (*d* = 0.8) required 29 observations (6 participants). Our cross-sectional sample comprised 1,517 observations from 157 participants (*M* = 9.67 observations per participant), while the longitudinal sample included 1,047 observations from 137 participants (*M* = 7.65 observations per participant). These sample sizes exceeded a priori requirements, yielding > 99% power cross-sectionally and longitudinally for small effects (*d* = 0.2) and > 99% power for medium to large effects (*d*≥ 0.5), aligning with multilevel model considerations outlined by Arend and Schäfer (2019).

## Results

### Participant Characteristics

Baseline demographic and clinical characteristics of participants are presented in Table 1. The sample comprised 157 adolescents, with 121 participants diagnosed with ongoing MDD and 36 diagnosed with previous but not ongoing MDD at baseline (*M*_age_ = 15.54, *SD* = 1.63; 71.34% female; 66.88% White; 13.38% Biracial; 9.55% Black, 8.92% Asian, 0.64% declined to answer, 0.64% Pacific Islander). All participants completed a series of laboratory research visits in Characterization (*N* = 157, *M*_visits_ = 8.64); some also completed weekly sessions of outpatient CBT (primarily BA) (*N* = 64, *M*_sessions_ = 7.11). All available data were used for each analysis. The full sample (*N* = 157) was utilized in concurrent analyses. Baseline correlational analyses included 148 participants with complete data on all relevant measures. Longitudinal models included between 140 and 142 participants, depending on the specific variables included in each model and availability of data for each participant.

**Table 1:** Demographic and clinical characteristics of participants

Variable	M (SD) or %; Range
Age	15.54 (1.61); 11–18
Sex (female)	71.34
Race	
White	66.88
Biracial	13.38
Black	9.55
Asian	8.92
Pacific Islander	0.64
Declined to answer	0.64
Ethnicity	
Non-Hispanic	0.90
Declined to answer	0.02
Clinical Measures at Baseline	
SHAPS	16.07 (6.92); 0–33
SCARED-SAD	7.88 (4.18); 0–14
SCARED-GAD	11.95 (4.88); 0–18

*SHAPS* Snaith-Hamilton Pleasure Scale, *SCARED-GAD* Screen for Child Anxiety Related Disorders-Generalized Anxiety Subscale, *SCARED-SAD* Screen for Child Anxiety Related Disorders-Social Anxiety Subscale. *N* = 148 for baseline analyses

**Fig. 1:**
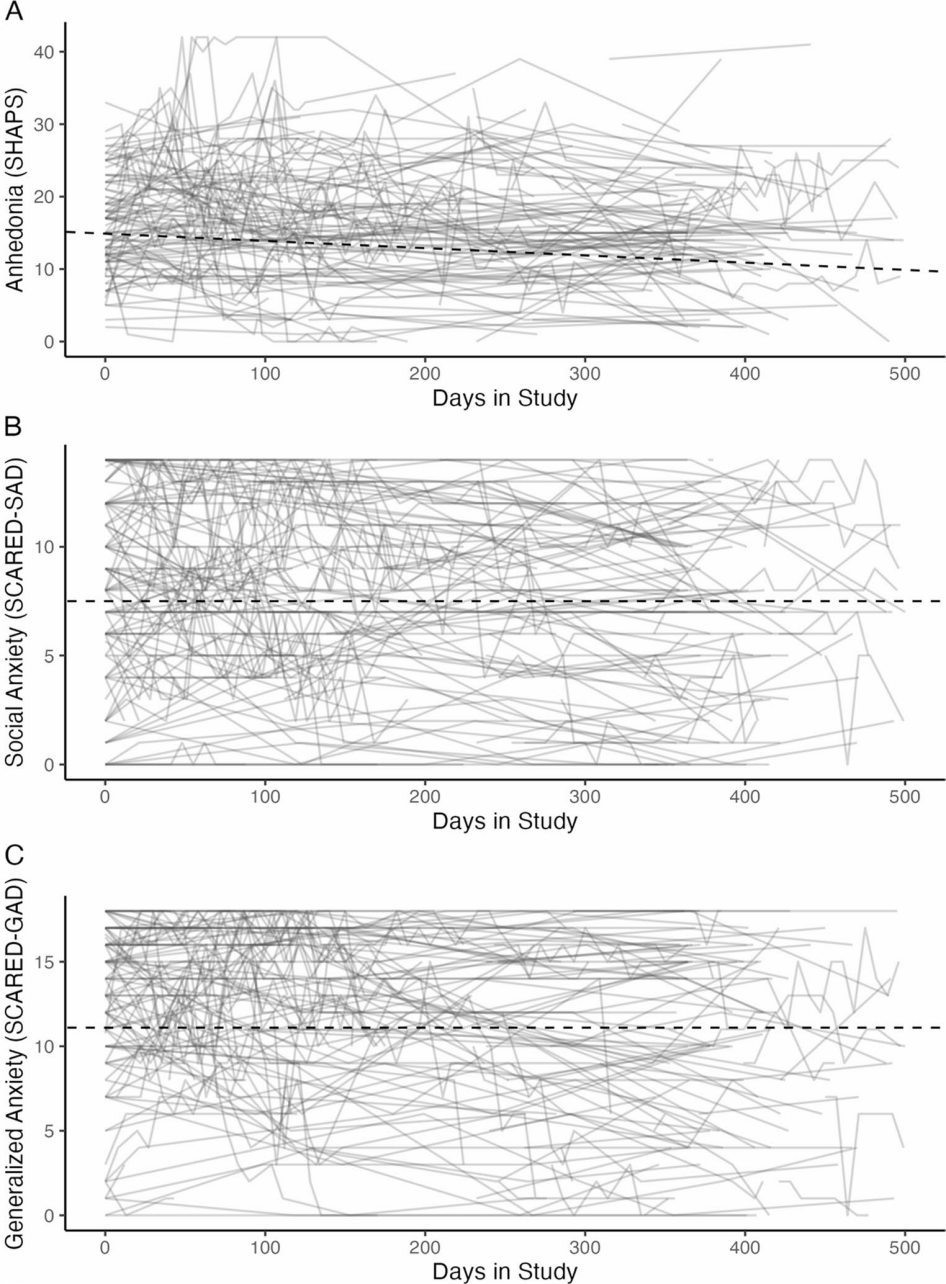
Anhedonia, social anxiety, and generalized anxiety scores across the first 500 days in study. Note. Each line represents an individual participant’s score over time, illustrating variability in trajectories. The black dashed line represents the MLM-predicted trajectory for each measure, derived from the equation using the intercept and slope for days in study

Baseline correlations between all measures are presented in Table 2. Additionally, Fig. 1 presents participants’ levels of anhedonia, social anxiety, and generalized anxiety across the first 500 days of the study, representing 58.80% of all data points. This timeframe was selected because participant attrition increased substantially after 500 days, resulting in fewer available data points beyond this period. Baseline means were calculated outside of the MLM model using all participants’ first available visit scores, which differ from those shown in Fig.1. It is important to note that some baseline data for questionnaires and diagnoses were missing for nine participants even though their IDs were included in the final dataset. As a result, the number of participants included in baseline analyses (*N* = 148) may differ slightly from the total sample size used in analyses (e.g., concurrent and longitudinal). The mean baseline SHAPS score across participants scores was 16.07; the SHAPS does not have established clinical cutoffs. An initial MLM testing SHAPS score as a function of the number of days in the study indicated that overall, the sample showed a decrease in anhedonia over time (*b* = − 0.003, *p* <.001). The mean baseline SCARED social anxiety subscale score was 7.88, with ≥ 8 being a clinically-significant threshold; the mean baseline SCARED generalized anxiety subscale score was 11.95, with ≥ 9 being a clinically significant threshold (Birmaher et al., 1997). Initial MLMs testing SCARED social anxiety and generalized anxiety scores as a function of the number of days in the study indicated that overall, the sample showed decreases in both social anxiety (*b* = − 0.001, *p* =.002) and generalized anxiety (*b* = − 0.002, *p* <.001) over time.

**Table 2:** Baseline pearson correlations for Anhedonia, Social, and generalized anxiety

Variable	1	2	3
1. SHAPS	-	-	-
2. SCARED-SAD	0.21*	-	-
3. SCARED-GAD	0.19*	0.54***	-

*p <.05. **p <.01. *** *p* <.001. Some baseline data were missing for nine participants, resulting in a slightly smaller sample size for these analyses (*N* = 148) than the full dataset (*N* = 157)

To explore whether baseline depression influenced associations between symptoms, we conducted exploratory Pearson correlations separately for participants with a previous (remitted) diagnosis of MDD (*n* = 36) and those with ongoing MDD (*n* = 121). Among participants with ongoing MDD, generalized and social anxiety symptoms were moderately correlated (*r* =.49, *p* <.001), but neither was significantly associated with anhedonia (SAD: *r* =.01, *p* =.92; GAD: *r* = −.04, *p* =.65). In contrast, within the previous MDD group, both generalized and social anxiety symptoms were significantly correlated with anhedonia (SAD: *r* =.19, *p* =.023; GAD: *r* =.21, *p* =.013), and GAD and SAD remained strongly related (*r* =.54, *p* <.001). A Fisher’s *r*-to-*z* comparison indicated that the association between GAD and anhedonia was significantly stronger in the previous MDD group than in the ongoing group (*z* = −2.12, *p* =.034). Differences in SAD–anhedonia correlations did not reach statistical significance (*p* =.13). These findings suggest potential variation in symptom relationships depending on depression status, though they were not guided by a priori hypotheses and should be interpreted with caution given the small sample size of the previous MDD group. To further contextualize these findings, we examined the baseline prevalence of anxiety disorders in the sample. Anxiety disorders were classified as either previous (lifetime) or ongoing (current). For social anxiety disorder, three participants were missing diagnostic data. Of the remaining 154 participants, 74 did not meet criteria for either previous or ongoing SAD, while 80 met criteria for previous SAD—79 of whom also met criteria for ongoing SAD. For generalized anxiety disorder (GAD), two participants were missing data. Of the remaining 155, 41 did not meet criteria for either previous or ongoing GAD, and 114 met criteria for both previous and ongoing GAD.

### Concurrent Associations

Full results for all models testing concurrent associations are presented in Table 3. As hypothesized, anhedonia was significantly associated with both social anxiety (*b* = 0.46, *p* =.002) and generalized anxiety (*b* = 0.37, *p* =.002) within time points.

### Temporal Associations

Full results for all models testing temporal associations are presented in Table 3. First, anhedonia, social anxiety, and generalized anxiety all showed significant auto-correlations from t-1 to t. Above and beyond these effects, as hypothesized, anhedonia at t-1 significantly predicted change in social anxiety from t-1 to t (*b* = 0.04, *p* =.008), such that within participants, higher-than-average levels of anhedonia predicted subsequent increases in social anxiety, whereas lower-than-average levels of anhedonia predicted subsequent decreases in social anxiety. However, contrary to expectations, anhedonia at t-1 did not predict change in generalized anxiety from t-1 to t (*b* = 0.03, *p* =.129). Likewise, social anxiety at t-1 did not predict change in anhedonia from t-1 to t (*b* = − 0.05, *p* =.671), and generalized anxiety at t-1 did not predict change in anhedonia from t-1 to t (*b* = 0.06, *p* =.458).

**Table 3:** Results of multilevel models testing Temporal associations

Social Anxiety Predicting Anhedonia				
Predictor	Unstd. coeff.	*SE*	*t*	*p* value
Intercept (Anhedonia_t_) (β_0j_)	14.89	0.65	23.02	0.002
Social Anxiety_t−1_ (β_1j_)	−0.05	0.11	−0.43	0.671
Anhedonia_t−1_ (β_2j_)	0.30	0.04	7.09	0.002
Days Between t and t-1 (β_3j_)	−0.01	0.00	−1.12	0.293
Generalized Anxiety Predicting Anhedonia				
Intercept (Anhedonia_t_) (β_0j_)	16.01	0.62	26.01	0.002
Generalized Anxiety_t−1_ (β_1j_)	0.06	0.07	0.79	0.458
Anhedonia_t−1_ (β_2j_)	0.28	0.04	6.87	0.002
Days Between t and t-1 (β_3j_)	−0.01	0.00	−1.15	0.293
Anhedonia Predicting Social Anxiety				
Intercept (Soc. Anx._t_) (β_0j_)	7.50	0.33	22.52	0.002
Anhedonia_t−1_ (β_1j_)	0.04	0.014	2.83	0.008
Social Anxiety_t−1_ (β_2j_)	0.26	0.05	5.61	0.002
Days Between t and t-1 (β_3j_)	−0.00	0.00	−1.49	0.187
Anhedonia Predicting Generalized Anxiety				
Intercept (Gen. Anx._t_) (β_0j_)	11.10	0.43	26.08	0.002
Anhedonia_t−1_ (β_1j_)	0.03	0.02	1.71	0.129
Generalized Anxiety_t−1_ (β_2j_)	0.24	0.05	5.17	0.002
Days Between t and t-1 (β_3j_)	−0.00	0.00	−1.19	0.293

## Discussion

The present study aimed to bridge gaps in the literature by exploring bidirectional relations between anhedonia and anxiety symptoms in adolescent MDD. We sought to comprehensively investigate both concurrent and temporal links of anhedonia with social anxiety and generalized anxiety symptoms at the within-person level. Consistent with our first hypothesis, anhedonia was positively associated with both social anxiety and generalized anxiety symptoms concurrently. Temporally, anhedonia emerged as a predictor of within-person change in social but not generalized anxiety at the subsequent time point, partially supporting our second hypothesis. That is, anhedonia was a unique temporal driver of social anxiety. This suggests that in adolescents with MDD, fluctuations in anhedonia may modulate the course of social anxiety symptoms across time, underscoring putative mechanistic links between anhedonia and social anxiety.

Notably, anhedonia predicted changes in social anxiety but not generalized anxiety. One potential explanation for this pattern is that unlike generalized anxiety, social anxiety is inherently tethered to reward-related processes. Social interactions involve reward anticipation, motivation, and learning (Kupferberg et al., 2016), making them especially relevant to anhedonia (Barkus & Badcock 2019). Adolescence is a period when peer acceptance and social belonging are critical to well-being (Marston et al., 2010); Sisk & Gee, (2022). During this time, social stressors become particularly salient, and the quality of social relationships plays a pivotal role in shaping mental health outcomes. By contrast, adolescents with diminished motivation and pleasure in response to social experiences may withdraw due to lowered expectations of enjoyment or a perceived inability to engage effectively (Watson et al., 2020). Over time, this reduced engagement may limit opportunities for positive reinforcement and social skill development, compounding both social anxiety and depression symptoms. In contrast, generalized anxiety disorder (GAD) is characterized by excessive, uncontrollable worry across multiple domains, including academic performance, health, and future uncertainty, rather than being narrowly tied to social contexts (Rowa et al., 2017). Although reward deficits might indirectly influence GAD symptoms—for instance, by dampening positive emotions (Eisner et al., 2009)—the primary characteristics of GAD may be more closely tied to broader cognitive processes than to specific reward-related dysfunction (Barkus & Badcock 2019).

Positive and negative social interactions can have profound effects on self-esteem, social competence, and overall well-being in early development. Anhedonia may reduce the drive to engage in positive social experiences (Watson et al., 2020), decreasing opportunities for connection and fostering feelings of alienation or inadequacy (Wittchen et al., 2000). Conversely, positive social relationships can serve as protective factors by promoting resilience and buffering against internalizing symptoms (Haddow et al., 2021); Kochel et al., 2017); Narr et al., (2019). Recognizing that social withdrawal and avoidance are core features of both social anxiety and depression, disruptions in interpersonal relationships during adolescence may contribute to the reciprocal associations between these conditions. Such dynamics have been explained through the lenses of Sensitivity Shift Theory (SST; Richey et al., 2019) and Reward Devaluation Theory (RDT; Winer & Salem, 2016). Sensitivity Shift Theory (SST) presents an integrated model addressing positive affect and motivational deficits in individuals with SAD. Social anhedonia refers to a trait-like disinterest in and absence of pleasure derived specifically from social interactions, and according to SST, a subset of youth who exhibit heightened sensitivity to both positive and negative social contexts may develop social anxiety through social anhedonia-driven avoidance. Reward Devaluation Theory (Winer & Salem, 2016) proposes that a subset of individuals with depression devalue and actively avoid positive or rewarding stimuli due to repeated associations with negative outcomes or potential for disappointment. In this way, positive experiences may become internally threatening, fostering avoidance of social situations that might otherwise be rewarding. Given the relatively stable nature of anhedonia throughout development (Bennik et al., 2014); Conway et al., (2017), reward-related aberrations may manifest as early risk factors that drive and maintain social anxiety symptoms through avoidance and negative reinforcement (Barkus, 2021); Taylor et al., (2022).

The unidirectional relationship from anhedonia to social anxiety raises important considerations. In adolescents with MDD, it is possible that reward dysfunction precedes and drives the emergence and maintenance of social anxiety symptoms, rather than vice versa. Notably, just over half of the current sample met criteria for both previous and ongoing SAD, suggesting chronicity for many but also variability in onset and course. During adolescence—a period in which peer rejection is acutely felt and social rewards are paramount (Somerville, (2013); (Lynn Mulvey et al. 2017)—this withdrawal may heighten fears of negative evaluation and reinforce avoidance behaviors, fueling social anxiety symptoms over time. Social anxiety involves a fundamental conflict between approach and avoidance tendencies (APA, 2013). While adolescents with social anxiety may deeply desire social connection, the fear of embarrassment and negative evaluation can create a paradox (Alden & Taylor, 2010); Caouette & Guyer, (2014). That is, they may deeply crave social acceptance yet simultaneously believe they are undeserving or incapable of achieving it. This internal conflict can lead to avoidance of social interactions despite a strong underlying desire for connection, reinforcing social anxiety and negative self-perceptions over time. Considering the heterogeneity of depression in adolescence (Chahal et al., 2020; Rice et al., 2019); (Thapar et al., 2022); Yaroslavsky et al., (Yaroslavsky et al. 2013), anhedonia may not affect all adolescents with MDD in the same way. For instance, those with comorbid SAD may experience more entrenched reward-related deficits than those without. Emerging evidence suggests that certain facets of reward-processing (i.e., sensitivity, motivation, learning; motivation; (Boyle et al., 2023) may contribute to distinct clinical presentations (Borsini et al., 2020); (Winer et al., 2019). Future work should investigate how subtypes of anhedonia (e.g., consummatory, anticipatory, learning; (Craske et al., 2016) manifest across different internalizing disorders and whether specific deficits in reward processing confer differential risk for specific forms of anxiety (i.e., social or generalized anxiety) compared to depression.

Exploratory analyses from the current study also revealed that baseline associations between anxiety and anhedonia differed based on MDD status, i.e., previous vs. ongoing. Specifically, the correlation between generalized anxiety symptoms and anhedonia was significantly stronger in adolescents with a previous (remitted) diagnosis of MDD compared to those with ongoing MDD. This finding, while post hoc and limited by a relatively small subgroup sample size, suggests that the nature of symptom relationships may shift along with depressive symptomatology. These preliminary results highlight the importance of considering diagnostic status when examining symptom dynamics and suggest that remission may alter the strength or salience of reward-related processes in relation to anxiety.

Beyond depression status, the co-occurrence of MDD and anxiety disorders at baseline in this study may have further shaped the symptom relationships observed over time. Given the high rates of comorbidity between these conditions, anxiety symptoms may contribute to persistent anhedonia, even among adolescents whose depressive symptoms have remitted. Moreover, the distinction between previous and ongoing MDD, in combination with anxiety disorder status, may shape the strength and direction of symptom interactions in ways not fully captured in this study. Although this study did not explicitly test these interactions, our findings underscore the value of a dimensional, symptom-based framework for tracking fluctuations in anhedonia and anxiety over time. Future research may benefit from simultaneously modeling changes in diagnostic status and symptom severity to better capture the dynamic nature of comorbid internalizing symptoms during adolescence.

Because our study focused on adolescents with MDD, anhedonia may have played a more central role in shaping secondary social anxiety symptoms—that is, symptoms emerging in the context of depression—whereas in primary social anxiety disorder (SAD), reward processing may remain intact but be disrupted by avoidance tendencies. This distinction may help explain why anhedonia predicted social anxiety symptoms over time, but not vice versa. Our diagnostic data revealed that most participants with SAD and GAD at baseline met criteria for both previous and ongoing diagnoses, reflecting high comorbidity and chronicity. These patterns suggest that in the context of adolescent MDD, comorbid anxiety may reflect a more severe or entrenched symptom profile, in which anhedonia exerts a stronger influence on anxiety symptom trajectories. Thus, while anhedonia may broadly contribute to internalizing symptoms, its effects on anxiety may be more domain-specific—particularly for social anxiety, given the critical role of social reward processing in maintaining fear and avoidance. Future research should investigate whether these patterns differ in adolescents with primary anxiety disorders, where SAD or GAD are the principal presenting concerns, rather than disorders co-occurring with or following depression.

### Clinical Implications

In light of our findings, there is a need for integrative approaches that target anhedonia in adolescents. Recent randomized clinical trials focusing on increasing positive affect and reward sensitivity in adult populations have demonstrated promising results in reducing stress, anxiety, and depression, surpassing the efficacy of traditional cognitive-behavioral interventions that primarily target negative affect (e.g., Craske et al., 2019, 2023); (McMakin et al., 2011); Taylor et al., (2017a, 2017b, 2024). While these interventions show promise, fewer studies have systematically tested similar approaches in adolescents. Emerging research suggests that interventions emphasizing positive emotional experiences—such as gratitude-based exercises, behavioral activation, and mindfulness—may be particularly beneficial in both clinical and school-based settings (Klos et al., 2020); Tejada-Gallardo et al., (Tejada-Gallardo et al. 2020); Owens & Waters, 2020). Even so, further research is needed to determine how these approaches can be optimally adapted for and implemented in youth populations. Given the distinct developmental challenges of adolescence, interventions tailored to enhance positive affect may serve as protective factors against stress while promoting mental health and resilience (Gilbert, 2012; Kansky et al., 2016); Sewart et al., 2019). Future work should focus on modifying and implementing reward-focused interventions in adolescent populations, identifying mechanisms of change, and evaluating their efficacy in improving co-occurring anxiety and depression symptoms across settings.

### Limitations

One significant limitation of this study is that data collection occurred partly during the COVID-19 pandemic. A multidimensional stressor of unprecedented scale (Gruber et al., 2021), this context likely precipitated interpersonal stress, reflecting atypical developmental trajectories and potentially confounding self-reports of anxiety and depression severity (Magson et al., 2021). Consequently, the contextual influence of the pandemic on our results may limit generalizability to otherwise typical adolescent experiences. Additionally, the reliance on self-report measures may introduce biases. Future studies may benefit from incorporating complementary levels of measurement, such as neurophysiological indices (e.g., heart rate, cortisol levels, neuroimaging) and behavioral assessments (e.g., decision-making tasks, social interaction paradigms, clinician-rated measures), as these could be more informative in identifying mechanistic links between anhedonia and social anxiety, where longitudinal associations were observed. Further, investigating anhedonic and diminished positive experiences across multiple facets of hedonic function, such as anticipatory pleasure, consummatory pleasure, and motivation for reward (Rizvi et al.,(Rizvi et al. 2015), could provide a more comprehensive understanding of its role in the development and maintenance of social anxiety and depression. While our findings are interpreted within a framework of secondary social anxiety symptoms emerging in the context of MDD, we did not have data on the temporal onset of comorbid conditions. As such, we use the term “secondary” to reflect the structure of the sample—adolescents with MDD—rather than to indicate diagnostic primacy. Future studies that systematically assess onset order could clarify how the sequencing of depression and anxiety influences symptom dynamics and developmental trajectories. Finally, while findings suggest a temporal role of anhedonia predicting social anxiety, replication in larger samples is needed to derive reliable clinical implications.

## Conclusions

We utilized a longitudinal sample of adolescents with ongoing or previous MDD followed longitudinally, allowing for a comprehensive understanding of clinical outcomes during a critical developmental period. Collectively, these results suggest that anhedonia may play a significant role in concurrent social anxiety and generalized anxiety symptoms in adolescent MDD, while also uniquely predicting social anxiety across time. These findings contribute to a growing body of literature on anhedonia in adolescent depression and as a transdiagnostic mechanism across mood and anxiety disorders. Additional research is necessary to replicate and extend the findings and to further inform interventions targeting emotional disorders in adolescents.

## Supplementary Information

Below is the link to the electronic supplementary material.

Supplementary Material 1

## Data Availability

Data are publicly available at: https://github.com/transatlantic-comppsych/CATD-study. Code used for analyses are available from the corresponding author upon request.

## References

[CR1] Adams, G. C., Balbuena, L., Meng, X., & Asmundson, G. J. G. (2016). When social anxiety and depression go together: A population study of comorbidity and associated consequences. *Journal of Affective Disorders*, *206*, 48–54. 10.1016/j.jad.2016.07.031 27466742 10.1016/j.jad.2016.07.031

[CR2] Aderka, I. M., Hofmann, S. G., Nickerson, A., Hermesh, H., Gilboa-Schechtman, E., & Marom, S. (2012). Functional impairment in social anxiety disorder. *Journal of Anxiety Disorders*, *26*(3), 393–400. 10.1016/j.janxdis.2012.01.003 22306132 10.1016/j.janxdis.2012.01.003

[CR3] Alden, L. E., & Taylor, C. T. (2010). Interpersonal processes in social anxiety disorder. *Interpersonal processes in the anxiety disorders: Implications for Understanding psychopathology and treatment* (pp. 125–152). American Psychological Association. 10.1037/12084-005

[CR4] American Psychiatric Association (2013). Diagnostic and statistical manual of mental disorders (5th ed.). 10.1176/appi.books.9780890425596

[CR5] American Psychiatric Association (2022). Diagnostic and statistical manual of mental disorders (5th ed., text rev.). 10.1176/appi.books.9780890425787

[CR6] Arend, M. G., & Schäfer, T. (2019). Statistical power in two-level models: A tutorial based on Monte Carlo simulation. *Psychological Methods*, *24*(1), 1–19. 10.1037/met0000195 30265048 10.1037/met0000195

[CR7] Auerbach, R. P., Pagliaccio, D., & Kirshenbaum, J. S. (2022). Anhedonia and suicide. *Current Topics in Behavioral Neurosciences*, *58*, 443–464. 10.1007/7854_2022_358 35435645 10.1007/7854_2022_358

[CR8] Bakker, J. M., Goossens, L., Lange, I., Michielse, S., Schruers, K., Lieverse, R., Marcelis, M., van Amelsvoort, T., van Os, J., Myin-Germeys, I., & Wichers, M. (2017). Real-life validation of reduced reward processing in emerging adults with depressive symptoms. *Journal of Abnormal Psychology*, *126*(6), 713–725. 10.1037/abn0000294 28782974 10.1037/abn0000294

[CR9] Barkus, E. (2021). The effects of anhedonia in social context. *Current Behavioral Neuroscience Reports*, *8*(3), 77–89. 10.1007/s40473-021-00232-x

[CR10] Barkus, E., & Badcock, J. C. (2019). A transdiagnostic perspective on social anhedonia. *Frontiers in Psychiatry*, *10*, 216. 10.3389/fpsyt.2019.00216 31105596 10.3389/fpsyt.2019.00216 PMC6491888

[CR11] Bennik, E. C., Nederhof, E., Ormel, J., & Oldehinkel, A. J. (2014). Anhedonia and depressed mood in adolescence: Course, stability, and reciprocal relation in the TRAILS study. *European Child & Adolescent Psychiatry*, *23*(7), 579–586. 10.1007/s00787-013-0481-z 24154568 10.1007/s00787-013-0481-z

[CR12] Birmaher, B., Khetarpal, S., Brent, D., Cully, M., Balach, L., Kaufman, J., & Neer, S. M. (1997). The screen for child anxiety related emotional disorders (SCARED): Scale construction and psychometric characteristics. *Journal of the American Academy of Child and Adolescent Psychiatry,**36*(4), 545–553. 9100430 10.1097/00004583-199704000-00018

[CR13] Borsini, A., Wallis, A. S. J., Zunszain, P., Pariante, C. M., & Kempton, M. J. (2020). Characterizing anhedonia: A systematic review of neuroimaging across the subtypes of reward processing deficits in depression. *Cognitive, Affective & Behavioral Neuroscience,**20*(4), 816–841. 10.3758/s13415-020-00804-6 10.3758/s13415-020-00804-6 PMC7395022 32472419

[CR14] Boyle, C. C., Bower, J. E., Eisenberger, N. I., & Irwin, M. R. (2023). Stress to inflammation and anhedonia: Mechanistic insights from preclinical and clinical models. *Neuroscience and Biobehavioral Reviews*, *152*, 105307. 10.1016/j.neubiorev.2023.105307 37419230 10.1016/j.neubiorev.2023.105307 PMC12273880

[CR15] Brown, T. A., Chorpita, B. F., & Barlow, D. H. (1998). Structural relationships among dimensions of the DSM-IV anxiety and mood disorders and dimensions of negative affect, positive affect, and autonomic arousal. *Journal of Abnormal Psychology*, *107*(2), 179–192. 10.1037/0021-843x.107.2.179 9604548 10.1037//0021-843x.107.2.179

[CR16] Caouette, J. D., & Guyer, A. E. (2014). Gaining insight into adolescent vulnerability for social anxiety from developmental cognitive neuroscience. *Developmental Cognitive Neuroscience*, *8*, 65–76. 10.1016/j.dcn.2013.10.003 24239049 10.1016/j.dcn.2013.10.003 PMC3960349

[CR17] Chahal, R., Gotlib, I. H., & Guyer, A. E. (2020). Research review: Brain network connectivity and the heterogeneity of depression in adolescence – A precision mental health perspective. *Journal of Child Psychology and Psychiatry and Allied Disciplines,**61*(12), 1282–1298. 10.1111/jcpp.13250 32458453 10.1111/jcpp.13250 PMC7688558

[CR18] Champely, S. (2020). pwr: Basic functions for power analysis (Version 1.3-0) RpackageR packageRpackage. Retrieved from https://github.com/heliosdrm/pwr

[CR19] Chorpita, B. F., & Weisz, J. R. (2009). Match-ADTC: Modular approach to therapy for children with anxiety, depression, trauma, or conduct problems. PracticeWise.

[CR20] Clark, L. A., & Watson, D. (1991). Tripartite model of anxiety and depression: Psychometric evidence and taxonomic implications. *Journal of Abnormal Psychology*, *100*(3), 316–336. 10.1037/0021-843x.100.3.316 1918611 10.1037//0021-843x.100.3.316

[CR21] Cohen, J. (1988). Statistical Power Analysis for the Behavioral Sciences (2nd ed.). Routledge. 10.4324/9780203771587

[CR22] Conway, C. C., Zinbarg, R. E., Mineka, S., & Craske, M. G. (2017). Core dimensions of anxiety and depression change independently during adolescence. *Journal of Abnormal Psychology*, *126*(2), 160–172. 10.1037/abn0000222 28192011 10.1037/abn0000222 PMC5308556

[CR23] Corsano, P., Majorano, M., & Champretavy, L. (2006). Psychological well-being in adolescence: The contribution of interpersonal relations and experience of being alone. *Adolescence*, *41*(162), 341–353. 16981621

[CR24] Craske, M. G., Meuret, A. E., Ritz, T., Treanor, M., & Dour, H. J. (2016). Treatment for anhedonia: A neuroscience driven approach. *Depression and Anxiety,**33*(10), 927–938. 10.1002/da.22490 27699943 10.1002/da.22490

[CR25] Craske, M. G., Meuret, A. E., Ritz, T., Treanor, M., Dour, H., & Rosenfield, D. (2019). Positive affect treatment for depression and anxiety: A randomized clinical trial for a core feature of anhedonia. *Journal of Consulting and Clinical Psychology*, *87*(5), 457–471. 10.1037/ccp0000396 30998048 10.1037/ccp0000396

[CR26] Craske, M. G., Meuret, A. E., Echiverri-Cohen, A., Rosenfield, D., & Ritz, T. (2023). Positive affect treatment targets reward sensitivity: A randomized controlled trial. *Journal of Consulting and Clinical Psychology*, *91*(6), 350–366. 10.1037/ccp0000805 36892884 10.1037/ccp0000805 PMC10213148

[CR27] Cuijpers, P., Karyotaki, E., Weitz, E., Andersson, G., Hollon, S. D., & van Straten, A. (2014). The effects of psychotherapies for major depression in adults on remission, recovery and improvement: A meta-analysis. *Journal of Affective Disorders*, *159*, 118–126. 10.1016/j.jad.2014.02.026 24679399 10.1016/j.jad.2014.02.026

[CR28] Cummings, C. M., Caporino, N. E., & Kendall, P. C. (2014). Comorbidity of anxiety and depression in children and adolescents: 20 years after. *Psychological Bulletin*, *140*(3), 816–845. 10.1037/a0034733 24219155 10.1037/a0034733 PMC4006306

[CR29] Dalrymple, K. L., & Zimmerman, M. (2007). Does comorbid social anxiety disorder impact the clinical presentation of principal major depressive disorder? *Journal of Affective Disorders*, *100*(1–3), 241–247. 10.1016/j.jad.2006.10.014 17188365 10.1016/j.jad.2006.10.014 PMC2547849

[CR30] Danneel, S., Nelemans, S., Spithoven, A., Bastin, M., Bijttebier, P., Colpin, H., Van Den Noortgate, W., Van Leeuwen, K., Verschueren, K., & Goossens, L. (2019). Internalizing problems in adolescence: Linking loneliness, social anxiety symptoms, and depressive symptoms over time. *Journal of Abnormal Child Psychology*, *47*(10), 1691–1705. 10.1007/s10802-019-00539-0 30937813 10.1007/s10802-019-00539-0

[CR31] Eisner, L. R., Johnson, S. L., & Carver, C. S. (2009). Positive affect regulation in anxiety disorders. *Journal of Anxiety Disorders*, *23*(5), 645–649. 10.1016/j.janxdis.2009.02.001 19278820 10.1016/j.janxdis.2009.02.001 PMC2847490

[CR32] Epkins, C. C., & Heckler, D. R. (2011). Integrating etiological models of social anxiety and depression in youth: Evidence for a cumulative interpersonal risk model. *Clinical Child and Family Psychology Review*, *14*(4), 329–376. 10.1007/s10567-011-0101-8 22080334 10.1007/s10567-011-0101-8

[CR33] Forbes, E. E., & Dahl, R. E. (2012). Research review: Altered reward function in adolescent depression: What, when and how? *Journal of Child Psychology and Psychiatry and Allied Disciplines,**53*(1), 3–15. 10.1111/j.1469-7610.2011.02477.x 22117893 10.1111/j.1469-7610.2011.02477.x PMC3232324

[CR34] Foulkes, L., & Blakemore, S. J. (2016). Is there heightened sensitivity to social reward in adolescence? *Current Opinion in Neurobiology*, *40*, 81–85. 10.1016/j.conb.2016.06.016 27420376 10.1016/j.conb.2016.06.016

[CR35] Fox, J., & Weisberg, S. (2019). An R companion to applied regression (3rd ed.). Sage. https://www.john-fox.ca/Companion/

[CR36] Franco, X., Saavedra, L. M., & Silverman, W. K. (2007). External validation of comorbid patterns of anxiety disorders in children and adolescents. *Journal of Anxiety Disorders*, *21*(5), 717–729. 10.1016/j.janxdis.2006.10.002 17095184 10.1016/j.janxdis.2006.10.002 PMC2692683

[CR37] Franken, I. H. A., Rassin, E., & Muris, P. (2007). The assessment of anhedonia in clinical and non-clinical populations: Further validation of the Snaith-Hamilton pleasure scale (SHAPS). *Journal of Affective Disorders*, *99*(1–3), 83–89. 10.1016/j.jad.2006.08.020 16996138 10.1016/j.jad.2006.08.020

[CR38] Gabbay, V., Johnson, A. R., Alonso, C. M., Evans, L. K., Babb, J. S., & Klein, R. G. (2015). Anhedonia, but not irritability, is associated with illness severity outcomes in adolescent major depression. *Journal of Child and Adolescent Psychopharmacology*, *25*(3), 194–200. 10.1089/cap.2014.0105 25802984 10.1089/cap.2014.0105 PMC4403015

[CR39] Galván, A. (2010). Adolescent development of the reward system. *Frontiers in Human Neuroscience*. 10.3389/neuro.09.006.2010. 4. 20179786 10.3389/neuro.09.006.2010 PMC2826184

[CR40] Galván, A. (2013). The teenage brain: Sensitivity to rewards. *Current Directions in Psychological Science*, *22*(2), 88–93. 10.1177/0963721413480859

[CR41] Garber, J., & Weersing, V. R. (2010). Comorbidity of anxiety and depression in youth: Implications for treatment and prevention. *Clinical Psychology: Science and Practice*, *17*(4), 293–306. 10.1111/j.1468-2850.2010.01221.x 21499544 10.1111/j.1468-2850.2010.01221.x PMC3074295

[CR42] Gilbert, K. E. (2012). The neglected role of positive emotion in adolescent psychopathology. *Clinical Psychology Review*, *32*(6), 467–481. 10.1016/j.cpr.2012.05.005 22710138 10.1016/j.cpr.2012.05.005

[CR43] Griffith, J. M., Long, E. E., Young, J. F., & Hankin, B. L. (2020). Co-occurring trajectories of depression and social anxiety in childhood and adolescence: Interactive effects of positive emotionality and domains of chronic interpersonal stress. *Journal of Abnormal Child Psychology*, *48*(6), 823–837. 10.1007/s10802-020-00634-7 32200465 10.1007/s10802-020-00634-7 PMC7251930

[CR44] Gruber, J., Prinstein, M. J., Clark, L. A., Rottenberg, J., Abramowitz, J. S., Albano, A. M., Aldao, A., Borelli, J. L., Chung, T., Davila, J., Forbes, E. E., Gee, D. G., Hall, G. C. N., Hallion, L. S., Hinshaw, S. P., Hofmann, S. G., Hollon, S. D., Joormann, J., Kazdin, A. E., Klein, D. N., & Weinstock, L. M. (2021). Mental health and clinical psychological science in the time of COVID-19: Challenges, opportunities, and a call to action. *The American Psychologist*, *76*(3), 409–426. 10.1037/amp0000707 32772538 10.1037/amp0000707 PMC7873160

[CR45] Guineau, M. G., Ikani, N., Rinck, M., Collard, R. M., van Eijndhoven, P., Tendolkar, I., Schene, A. H., Becker, E. S., & Vrijsen, J. N. (2023). Anhedonia as a transdiagnostic symptom across psychological disorders: A network approach. *Psychological Medicine*, *53*(9), 3908–3919. 10.1017/S0033291722000575 35348051 10.1017/S0033291722000575 PMC10317820

[CR46] Gupta, T., Eckstrand, K. L., & Forbes, E. E. (2024a). Annual research review: Puberty and the development of anhedonia–considering childhood adversity and inflammation. *Journal of Child Psychology and Psychiatry,**65*(4), 459–480. 10.1111/jcpp.13955 38391011 10.1111/jcpp.13955 PMC10939801

[CR47] Gupta, T., Seah, T. H. S., Eckstrand, K. L., Rengasamy, M., Horter, C., Silk, J., Jones, N., Ryan, N. D., Phillips, M. L., Haas, G., Nance, M., Lindenmuth, M., & Forbes, E. E. (2024b). Two-year trajectories of anhedonia in adolescents at transdiagnostic risk for severe mental illness: Association with clinical symptoms and brain-symptom links. *Journal of Psychopathology and Clinical Science,**133*(8), 618–629. 10.1037/abn0000938 39480330 10.1037/abn0000938 PMC11740312

[CR48] Haddow, S., Taylor, E. P., & Schwannauer, M. (2021). Positive peer relationships, coping and resilience in young people in alternative care: A systematic review. *Children and Youth Services Review,**122*,. 10.1016/j.childyouth.2020.105861

[CR49] Hamilton, J. L., Potter, C. M., Olino, T. M., Abramson, L. Y., Heimberg, R. G., & Alloy, L. B. (2016). The temporal sequence of social anxiety and depressive symptoms following interpersonal stressors during adolescence. *Journal of Abnormal Child Psychology,**44*(3), 495–509. 10.1007/s10802-015-0049-0 26142495 10.1007/s10802-015-0049-0 PMC4701637

[CR50] Jacobson, N. C., & Newman, M. G. (2017). Anxiety and depression as bidirectional risk factors for one another: A meta-analysis of longitudinal studies. *Psychological Bulletin,**143*(11), 1155–1200. 10.1037/bul0000111 28805400 10.1037/bul0000111

[CR51] Johnson, D., Dupuis, G., Piche, J., Clayborne, Z., & Colman, I. (2018). Adult mental health outcomes of adolescent depression: A systematic review. *Depression and Anxiety*, *35*(8), 700–716. 10.1002/da.22777 29878410 10.1002/da.22777

[CR52] Kalin, N. H. (2021). Anxiety, depression, and suicide in youth. *The American Journal of Psychiatry*, *178*(4), 275–279. 10.1176/appi.ajp.2020.21020186 33789454 10.1176/appi.ajp.2020.21020186

[CR53] Kansky, J., Allen, J. P., & Diener, E. (2016). Early adolescent affect predicts later life outcomes. *Applied Psychology: Health and Well-Being,**8*(2), 192–212. 27075545 10.1111/aphw.12068 PMC4931979

[CR54] Kashdan, T. B. (2004). The neglected relationship between social interaction anxiety and hedonic deficits: Differentiation from depressive symptoms. *Journal of Anxiety Disorders*, *18*(5), 719–730. 10.1016/j.janxdis.2003.08.001 15275949 10.1016/j.janxdis.2003.08.001

[CR55] Kashdan, T. B. (2007). Social anxiety spectrum and diminished positive experiences: Theoretical synthesis and meta-analysis. *Clinical Psychology Review*, *27*(3), 348–365. 10.1016/j.cpr.2006.12.003 17222490 10.1016/j.cpr.2006.12.003

[CR56] Kashdan, T. B., & Steger, M. F. (2006). Expanding the topography of social anxiety. An experience-sampling assessment of positive emotions, positive events, and emotion suppression. *Psychological Science*, *17*(2), 120–128. 10.1111/j.1467-9280.2006.01674.x 16466419 10.1111/j.1467-9280.2006.01674.x

[CR57] Kaufman, J., Birmaher, B., Brent, D., Rao, U., Flynn, C., Moreci, P., Williamson, D., & Ryan, N. (1997). Initial reliability and validity data. *Journal of the American Academy of Child and Adolescent Psychiatry*, *36*(7), 980–988. 10.1097/00004583-199707000-00021. Schedule for affective disorders and schizophrenia for school-age children-present and lifetime version (K-SADS-PL). 10.1097/00004583-199707000-00021 9204677

[CR58] Klos, L., Feil, K., Eberhardt, T., & Jekauc, D. (2020). Interventions to promote positive affect and physical activity in children, adolescents and young adults—a systematic review. *Sports*, *8*(2), 26. 10.3390/sports8020026 32093347 10.3390/sports8020026 PMC7076746

[CR59] Kochel, K. P., Bagwell, C. L., Ladd, G. W., & Rudolph, K. D. (2017). Do positive peer relations mitigate transactions between depressive symptoms and peer victimization in adolescence? *Journal of Applied Developmental Psychology*, *51*, 44–54. 10.1016/j.appdev.2017.04.003 29104337 10.1016/j.appdev.2017.04.003 PMC5667670

[CR60] Koo, T. K., & Li, M. Y. (2016). A guideline of selecting and reporting intraclass correlation coefficients for reliability research. *Journal of Chiropractic Medicine*, *15*(2), 155–163. 10.1016/j.jcm.2016.02.012 27330520 10.1016/j.jcm.2016.02.012 PMC4913118

[CR61] Koyuncu, A., İnce, E., Ertekin, E., & Tükel, R. (2019). Comorbidity in social anxiety disorder: Diagnostic and therapeutic challenges. *Drugs in Context,**8*, Article 212573. 10.7573/dic.212573 30988687 10.7573/dic.212573 PMC6448478

[CR62] Kupferberg, A., Bicks, L., & Hasler, G. (2016). Social functioning in major depressive disorder. *Neuroscience and Biobehavioral Reviews*, *69*, 313–332. 10.1016/j.neubiorev.2016.07.002 27395342 10.1016/j.neubiorev.2016.07.002

[CR63] Lamblin, M., Murawski, C., Whittle, S., & Fornito, A. (2017). Social connectedness, mental health and the adolescent brain. *Neuroscience and Biobehavioral Reviews*, *80*, 57–68. 10.1016/j.neubiorev.2017.05.010 28506925 10.1016/j.neubiorev.2017.05.010

[CR64] Langvik, E., & Borgen Austad, S. (2019). Psychometric properties of the Snaith–Hamilton pleasure scale and a facet-level analysis of the relationship between anhedonia and extraversion in a nonclinical sample. *Psychological Reports*, *122*(1), 360–375. 10.1177/0033294118756336 29490559 10.1177/0033294118756336

[CR65] Leventhal, A. M., Unger, J. B., Audrain-McGovern, J., Sussman, S., Volk, H. E., & Strong, D. R. (2015). Measuring anhedonia in adolescents: A psychometric analysis. *Journal of Personality Assessment,**97*(5), 506–514. 10.1080/00223891.2015.1029072 25893676 10.1080/00223891.2015.1029072 PMC4545400

[CR66] Leventhal, A. M., Cho, J., Stone, M. D., Barrington-Trimis, J. L., Chou, C. P., Sussman, S. Y., Riggs, N. R., Unger, J. B., Audrain-McGovern, J., & Strong, D. R. (2017). Associations between anhedonia and marijuana use escalation across mid-adolescence. *Addiction (Abingdon England)*, *112*(12), 2182–2190. 10.1111/add.13912 28623880 10.1111/add.13912 PMC5673572

[CR67] Liang, S., Wu, Y., Hanxiaoran, L., Greenshaw, A. J., & Li, T. (2022). Anhedonia in depression and schizophrenia: Brain reward and aversion circuits. *Neuropsychiatric Disease and Treatment,* 1385. 10.2147/NDT.S367839 35836582 10.2147/NDT.S367839 PMC9273831

[CR68] Long, E. E., Young, J. F., & Hankin, B. L. (2018). Temporal dynamics and longitudinal co-occurrence of depression and different anxiety syndromes in youth: Evidence for reciprocal patterns in a 3-year prospective study. *Journal of Affective Disorders*, *234*, 20–27. 10.1016/j.jad.2018.02.074 29522939 10.1016/j.jad.2018.02.074 PMC5895498

[CR69] Luckhardt, C., Mühlherr, A. M., Schütz, M., Jarczok, T. A., Jungmann, S. M., Howland, V., Veit, L., Althen, H., & Freitag, C. M. (2023). Reward processing in adolescents with social phobia and depression. *Clinical Neurophysiology,**150*, 205–215. 10.1016/j.clinph.2023.03.356 37104910 10.1016/j.clinph.2023.03.356

[CR70] Luking, K. R., Pagliaccio, D., Luby, J. L., & Barch, D. M. (2016). Reward processing and risk for depression across development. *Trends in Cognitive Sciences*, *20*(6), 456–468. 10.1016/j.tics.2016.04.002 27131776 10.1016/j.tics.2016.04.002 PMC4875800

[CR71] Lynn Mulvey, K., Boswell, C., & Zheng, J. (2017). Causes and consequences of social exclusion and peer rejection among children and adolescents. *Report on Emotional & Behavioral Disorders in Youth*, *17*(3), 71–75. 30100820 PMC6085085

[CR72] Magson, N. R., Freeman, J. Y. A., Rapee, R. M., Richardson, C. E., Oar, E. L., & Fardouly, J. (2021). Risk and protective factors for prospective changes in adolescent mental health during the COVID-19 pandemic. *Journal of Youth and Adolescence*, *50*(1), 44–57. 10.1007/s10964-020-01332-9 33108542 10.1007/s10964-020-01332-9 PMC7590912

[CR73] Marston, E. G., Hare, A., & Allen, J. P. (2010). Rejection sensitivity in late adolescence: Social and emotional sequelae. *Journal of Research on Adolescence,**20*(4), 959–982. 10.1111/j.1532-7795.2010.00675.x 21113326 10.1111/j.1532-7795.2010.00675.x PMC2990973

[CR74] McLaughlin, K. A., & King, K. (2015). Developmental trajectories of anxiety and depression in early adolescence. *Journal of Abnormal Child Psychology*, *43*(2), 311–323. 10.1007/s10802-014-9898-1 24996791 10.1007/s10802-014-9898-1 PMC4286282

[CR75] McMakin, D. L., Siegle, G. J., & Shirk, S. R. (2011). Positive affect stimulation and sustainment (PASS) module for depressed mood: A preliminary investigation of treatment-related effects. *Cognitive Therapy and Research*, *35*(3), 217–226. 10.1007/s10608-010-9311-5 22140287 10.1007/s10608-010-9311-5 PMC3226735

[CR76] McMakin, D. L., Olino, T. M., Porta, G., Dietz, L. J., Emslie, G., Clarke, G., Wagner, K. D., Asarnow, J. R., Ryan, N. D., Birmaher, B., Shamseddeen, W., Mayes, T., Kennard, B., Spirito, A., Keller, M., Lynch, F. L., Dickerson, J. F., & Brent, D. A. (2012). Anhedonia predicts poorer recovery among youth with selective serotonin reuptake inhibitor treatment-resistant depression. *Journal of the American Academy of Child and Adolescent Psychiatry*, *51*(4), 404–411. 10.1016/j.jaac.2012.01.011 22449646 10.1016/j.jaac.2012.01.011 PMC3536476

[CR77] Melton, T. H., Croarkin, P. E., Strawn, J. R., & Mcclintock, S. M. (2016). Comorbid anxiety and depressive symptoms in children and adolescents: A systematic review and analysis. *Journal of Psychiatric Practice*, *22*(2), 84–98. 10.1097/PRA.0000000000000132 27138077 10.1097/PRA.0000000000000132 PMC6267783

[CR78] Nakonezny, P. A., Morris, D. W., Greer, T. L., Byerly, M. J., Carmody, T. J., Grannemann, B. D., Bernstein, I. H., & Trivedi, M. H. (2015). Evaluation of anhedonia with the Snaith-Hamilton pleasure scale (SHAPS) in adult outpatients with major depressive disorder. *Journal of Psychiatric Research*, *65*, 124–130. 10.1016/j.jpsychires.2015.03.010 25864641 10.1016/j.jpsychires.2015.03.010 PMC7505238

[CR79] Narr, R. K., Allen, J. P., Tan, J. S., & Loeb, E. L. (2019). Close friendship strength and broader peer group desirability as differential predictors of adult mental health. *Child Development*, *90*(1), 298–313. 10.1111/cdev.12905 28832975 10.1111/cdev.12905 PMC5821600

[CR80] Nock, M. K., & Kazdin, A. E. (2002). Examination of affective, cognitive, and behavioral factors and suicide-related outcomes in children and young adolescents. *Journal of Clinical Child and Adolescent Psychology: The Official Journal for the Society of Clinical Child and Adolescent Psychology, American Psychological Association, Division 53,**31*(1), 48–58. 10.1207/S15374424JCCP3101_07 11845650 10.1207/S15374424JCCP3101_07

[CR81] Nock, M. K., Hwang, I., Sampson, N. A., & Kessler, R. C. (2010). Mental disorders, comorbidity and suicidal behavior: Results from the National Comorbidity Survey Replication. *Molecular Psychiatry,**15*(8), 868–876. 10.1038/mp.2009.29 19337207 10.1038/mp.2009.29 PMC2889009

[CR82] O’Neil, K. A., Podell, J. L., Benjamin, C. L., & Kendall, P. C. (2010). Comorbid depressive disorders in anxiety-disordered youth: Demographic, clinical, and family characteristics. *Child Psychiatry and Human Development*, *41*(3), 330–341. 10.1007/s10578-009-0170-9 20066489 10.1007/s10578-009-0170-9

[CR83] Oberle, E., Schonert-Reichl, K. A., & Thomson, K. C. (2010). Understanding the link between social and emotional well-being and peer relations in early adolescence: Gender-specific predictors of peer acceptance. *Journal of Youth and Adolescence*, *39*(11), 1330–1342. 10.1007/s10964-009-9486-9 20091211 10.1007/s10964-009-9486-9

[CR84] Ohayon, M. M., & Schatzberg, A. F. (2010). Social phobia and depression: Prevalence and comorbidity. *Journal of Psychosomatic Research*, *68*(3), 235–243. 10.1016/j.jpsychores.2009.07.018 20159208 10.1016/j.jpsychores.2009.07.018

[CR85] Owens, R. L., & Waters, L. (2020). What does positive psychology tell us about early intervention and prevention with children and adolescents? A review of positive psychological interventions with young people. *The Journal of Positive Psychology,**15*(5), 588–597. 10.1080/17439760.2020.1789706

[CR86] Pittig, A., Boschet, J. M., Glück, V. M., & Schneider, K. (2021). Elevated costly avoidance in anxiety disorders: Patients show little downregulation of acquired avoidance in face of competing rewards for approach. *Depression and Anxiety*, *38*(3), 361–371. 10.1002/da.23119 33258530 10.1002/da.23119

[CR87] Pizzagalli, D. A. (2022). Toward a better understanding of the mechanisms and pathophysiology of anhedonia: Are we ready for translation? *American Journal of Psychiatry,**179*(7), 458–469. 10.1176/appi.ajp.20220423 35775159 10.1176/appi.ajp.20220423 PMC9308971

[CR88] Prenoveau, J. M., Zinbarg, R. E., Craske, M. G., Mineka, S., Griffith, J. W., & Epstein, A. M. (2010). Testing a hierarchical model of anxiety and depression in adolescents: A tri-level model. *Journal of Anxiety Disorders*, *24*(3), 334–344. 10.1016/j.janxdis.2010.01.006 20171054 10.1016/j.janxdis.2010.01.006

[CR89] Rapee, R. M., Oar, E. L., Johnco, C. J., Forbes, M. K., Fardouly, J., Magson, N. R., & Richardson, C. E. (2019). Adolescent development and risk for the onset of social-emotional disorders: A review and conceptual model. *Behaviour Research and Therapy*, *123*, 103501. 10.1016/j.brat.2019.103501 31733812 10.1016/j.brat.2019.103501

[CR90] Raudenbush, S. W., & Congdon, R. T. (2021). *HLM 8: Hierarchical linear and nonlinear modeling*. Scientific Software International, Inc.

[CR91] Revelle, W. (2024). psych: Procedures for psychological, psychometric, and personality research (Version 2.4.12) [R package]. Northwestern University. Retrieved from https://CRAN.R-project.org/package=psych

[CR92] Rice, F., Riglin, L., Lomax, T., Souter, E., Potter, R., Smith, D. J., Thapar, A. K., & Thapar, A. (2019). Adolescent and adult differences in major depression symptom profiles. *Journal of Affective Disorders*, *243*, 175–181. 10.1016/j.jad.2018.09.015 30243197 10.1016/j.jad.2018.09.015

[CR93] Richey, J. A., Brewer, J. A., Sullivan-Toole, H., Strege, M. V., Kim-Spoon, J., White, S. W., & Ollendick, T. H. (2019). Sensitivity shift theory: A developmental model of positive affect and motivational deficits in social anxiety disorder. *Clinical Psychology Review*, *72*, 101756. 10.1016/j.cpr.2019.101756 31351312 10.1016/j.cpr.2019.101756

[CR94] Rizvi, S. J., Quilty, L. C., Sproule, B. A., Cyriac, A., Bagby, M., R., & Kennedy, S. H. (2015). Development and validation of the dimensional anhedonia rating scale (DARS) in a community sample and individuals with major depression. *Psychiatry Research*, *229*(1–2), 109–119. 10.1016/j.psychres.2015.07.062 26250147 10.1016/j.psychres.2015.07.062

[CR95] Roach, A. (2018). Supportive peer relationships and mental health in adolescence: An integrative review. *Issues in Mental Health Nursing*, *39*(9), 723–737. 10.1080/01612840.2018.1496498 30252560 10.1080/01612840.2018.1496498

[CR96] Rosenberg, B. M., Barnes-Horowitz, N. M., Zbozinek, T. D., & Craske, M. G. (2024). Reward processes in extinction learning and applications to exposure therapy. *Journal of Anxiety Disorders*, *106*, 102911. 10.1016/j.janxdis.2024.102911 39128178 10.1016/j.janxdis.2024.102911 PMC11384290

[CR97] Rowa, K., Waechter, S., Hood, H. K., & Antony, M. M. (2017). Generalized anxiety disorder. Psychopathology: History, Diagnosis, and Empirical Foundations, Third Edition, 149–186.

[CR98] RStudio Team (2024). RStudio: Integrated development environment for R [Computer software]. PBC. http://www.rstudio.com/

[CR99] Runyon, K., Chesnut, S. R., & Burley, H. (2018). Screening for childhood anxiety: A meta-analysis of the Screen for Child Anxiety Related Emotional Disorders. *Journal of Affective Disorders,**240*, 220–229. 10.1016/j.jad.2018.07.049 30081293 10.1016/j.jad.2018.07.049

[CR100] Sandman, C. F., & Craske, M. G. (2022). Psychological treatments for anhedonia. *Current Topics in Behavioral Neurosciences*, *58*, 491–513. 10.1007/7854_2021_291 34935116 10.1007/7854_2021_291

[CR101] Schwartz, K. T. G., Kryza-Lacombe, M., Liuzzi, M. T., Weersing, V. R., & Wiggins, J. L. (2019). Social and non-social reward: A preliminary examination of clinical improvement and neural reactivity in adolescents treated with behavioral therapy for anxiety and depression. *Frontiers in Behavioral Neuroscience*, *13*, 177. 10.3389/fnbeh.2019.00177 31551724 10.3389/fnbeh.2019.00177 PMC6736628

[CR102] Sewart, A. R., Zbozinek, T. D., Hammen, C., Zinbarg, R. E., Mineka, S., & Craske, M. G. (2019). Positive affect as a buffer between chronic stress and symptom severity of emotional disorders. *Clinical Psychological Science,**7*(5), 914–927. 10.1177/2167702619834576 31632843 10.1177/2167702619834576 PMC6800737

[CR103] Sisk, L. M., & Gee, D. G. (2022). Stress and adolescence: Vulnerability and opportunity during a sensitive window of development. *Current Opinion in Psychology*, *44*, 286–292. 10.1016/j.copsyc.2021.10.005 34818623 10.1016/j.copsyc.2021.10.005 PMC9007828

[CR104] Snaith, R. P., Hamilton, M., Morley, S., Humayan, A., Hargreaves, D., & Trigwell, P. (1995). A scale for the assessment of hedonic tone the Snaith-Hamilton pleasure scale. *The British Journal of Psychiatry,**167*(1), 99–103. 10.1192/bjp.167.1.99 7551619 10.1192/bjp.167.1.99

[CR105] Snijders, T. A. B., & Bosker, R. J. (2011). Multilevel analysis. An introduction to basic and advanced multilevel modeling. (2nd (1st edition 1999) ed.) SAGE Publications Inc.

[CR106] Somerville, L. H. (2013). The teenage brain: Sensitivity to social evaluation. *Current Directions in Psychological Science*, *22*(2), 121–127. 10.1177/0963721413476512 24761055 10.1177/0963721413476512 PMC3992953

[CR107] Spijker, J., Bijl, R. V., de Graaf, R., & Nolen, W. A. (2001). Determinants of poor 1-year outcome of DSM-III-R major depression in the general population: Results of the Netherlands mental health survey and incidence study (NEMESIS). *Acta Psychiatrica Scandinavica,**103*(2), 122–130. 10.1034/j.1600-0447.2001.103002122.x 11167315 10.1034/j.1600-0447.2001.103002122.x

[CR108] Stein, M. B., Roy-Byrne, P. P., Craske, M. G., Bystritsky, A., Sullivan, G., Pyne, J. M., Katon, W., & Sherbourne, C. D. (2005). Functional impact and health utility of anxiety disorders in primary care outpatients. *Medical Care*, *43*(12), 1164–1170. 10.1097/01.mlr.0000185750.18119.fd 16299426 10.1097/01.mlr.0000185750.18119.fd

[CR109] Taylor, C. T., Knapp, S. E., Bomyea, J. A., Ramsawh, H. J., Paulus, M. P., & Stein, M. B. (2017a). What good are positive emotions for treatment? Trait positive emotionality predicts response to cognitive behavioral therapy for anxiety. *Behaviour Research and Therapy*, *93*, 6–12. 10.1016/j.brat.2017.03.006 28342947 10.1016/j.brat.2017.03.006 PMC5627362

[CR110] Taylor, C. T., Lyubomirsky, S., & Stein, M. B. (2017b). Upregulating the positive affect system in anxiety and depression: Outcomes of a positive activity intervention. *Depression and Anxiety,**34*(3), 267–280. 10.1002/da.22593 28060463 10.1002/da.22593 PMC7266488

[CR111] Taylor, C. T., Hoffman, S. N., & Khan, A. J. (2022). Anhedonia in anxiety disorders. In D. A. Pizzagalli (Ed.), *Anhedonia: Preclinical, translational, and clinical integration* (pp. 201–218). Springer International Publishing.

[CR112] Taylor, C. T., Stein, M. B., Simmons, A. N., He, F., Oveis, C., Shakya, H. B., Sieber, W. J., Fowler, J. H., & Jain, S. (2024). Amplification of positivity treatment for anxiety and depression: A randomized experimental therapeutics trial targeting social reward sensitivity to enhance social connectedness. *Biological Psychiatry*, *95*(5), 434–443. 10.1016/j.biopsych.2023.07.024 37607657 10.1016/j.biopsych.2023.07.024 PMC12063735

[CR113] Tejada-Gallardo, C., Blasco-Belled, A., Torrelles-Nadal, C., & Alsinet, C. (2020). Effects of school-based multicomponent positive psychology interventions on well-being and distress in adolescents: A systematic review and meta-analysis. *Journal of Youth and Adolescence*, *49*(10), 1943–1960. 10.1007/s10964-020-01289-9 32683592 10.1007/s10964-020-01289-9

[CR114] Thapar, A., Eyre, O., Patel, V., & Brent, D. (2022). Depression in young people. *The Lancet*, *400*(10352), 617–631. 10.1016/S0140-6736(22)01012-1 10.1016/S0140-6736(22)01012-1 35940184

[CR115] Treadway, M. T., & Zald, D. H. (2011). Reconsidering anhedonia in depression: Lessons from translational neuroscience. *Neuroscience and Biobehavioral Reviews*, *35*(3), 537–555. 10.1016/j.neubiorev.2010.06.006 20603146 10.1016/j.neubiorev.2010.06.006 PMC3005986

[CR116] Vrieze, E., Pizzagalli, D. A., Demyttenaere, K., Hompes, T., Sienaert, P., de Boer, P., Schmidt, M., & Claes, S. (2013). Reduced reward learning predicts outcome in major depressive disorder. *Biological Psychiatry*, *73*(7), 639–645. 10.1016/j.biopsych.2012.10.014 23228328 10.1016/j.biopsych.2012.10.014 PMC3602158

[CR117] Walker, D. M., Bell, M. R., Flores, C., Gulley, J. M., Willing, J., & Paul, M. J. (2017). Adolescence and reward: Making sense of neural and behavioral changes amid the chaos. *The Journal of Neuroscience*, *37*(45), 10855–10866. 10.1523/JNEUROSCI.1834-17.2017 29118215 10.1523/JNEUROSCI.1834-17.2017 PMC5678018

[CR118] Watson, R., Harvey, K., McCabe, C., & Reynolds, S. (2020). Understanding anhedonia: A qualitative study exploring loss of interest and pleasure in adolescent depression. *European Child & Adolescent Psychiatry*, *29*(4), 489–499. 10.1007/s00787-019-01364-y 31270605 10.1007/s00787-019-01364-y PMC7103575

[CR119] Wechsler, D. (2011). *Wechsler abbreviated scale of Intelligence–Second edition (WASI-II)*. NCS Pearson.

[CR120] Wickham, H. (2016). Ggplot2: Elegant graphics for data analysis (2nd ed.) [PDF]. Springer International Publishing.

[CR121] Wickham, H., François, R., Henry, L., Müller, K., & Vaughan, D. (2023). dplyr: A grammar of data manipulation (Version 1.1.4) [R package]. Retrieved from https://dplyr.tidyverse.org

[CR122] Winer, E. S., & Salem, T. (2016). Reward devaluation: Dot-probe meta-analytic evidence of avoidance of positive information in depressed persons. *Psychological Bulletin*, *142*(1), 18–78. 10.1037/bul0000022 26619211 10.1037/bul0000022 PMC4688138

[CR123] Winer, E. S., Nadorff, M. R., Ellis, T. E., Allen, J. G., Herrera, S., & Salem, T. (2014). Anhedonia predicts suicidal ideation in a large psychiatric inpatient sample. *Psychiatry Research*, *218*(1–2), 124–128. 10.1016/j.psychres.2014.04.016 24774075 10.1016/j.psychres.2014.04.016

[CR124] Winer, E. S., Bryant, J., Bartoszek, G., Rojas, E., Nadorff, M. R., & Kilgore, J. (2017). Mapping the relationship between anxiety, anhedonia, and depression. *Journal of Affective Disorders*, *221*, 289–296. 10.1016/j.jad.2017.06.006 28668590 10.1016/j.jad.2017.06.006 PMC6080718

[CR125] Winer, E. S., Jordan, D. G., & Collins, A. C. (2019). Conceptualizing anhedonias and implications for depression treatments. *Psychology Research and Behavior Management,* 325–335. 10.2147/PRBM.S159260 31191054 10.2147/PRBM.S159260 PMC6521843

[CR126] Wittchen, H. U., Kessler, R. C., Pfister, H., Höfler, M., & Lieb, R. (2000). Why do people with anxiety disorders become depressed? A prospective-longitudinal community study. *Acta Psychiatrica Scandinavica*, *102*(s406), 14–23. 10.1111/j.0065-1591.2000.acp29-03.x 11131466

[CR127] Wong, S., Le, G. H., Phan, L., Rhee, T. G., Ho, R., Meshkat, S., Teopiz, K. M., Kwan, A. T. H., Mansur, R. B., Rosenblat, J. D., & McIntyre, R. S. (2024). Effects of anhedonia on health-related quality of life and functional outcomes in major depressive disorder: A systematic review and meta-analysis. *Journal of Affective Disorders*, *356*, 684–698. 10.1016/j.jad.2024.04.086 38657767 10.1016/j.jad.2024.04.086

[CR128] Yaroslavsky, I., Pettit, J. W., Lewinsohn, P. M., Seeley, J. R., & Roberts, R. E. (2013). Heterogeneous trajectories of depressive symptoms: Adolescent predictors and adult outcomes. *Journal of Affective Disorders*, *148*(2–3), 391–399. 10.1016/j.jad.2012.06.028 22963892 10.1016/j.jad.2012.06.028 PMC3654021

