# Correction: Temporal Predictions from Anhedonia To Anxiety in Adolescents with Major Depressive Disorder

**DOI:** 10.1007/s10802-025-01384-0

**Published:** 2025-10-09

**Authors:** Yannie D. Lee, Kenneth Towbin, Daniel S. Pine, Argyris Stringaris, Katharina Kircanski

**Affiliations:** 1 Emotion and Development Branch, National Institute of Mental Health, Bethesda, MD USA; 2 University College London, London, UK; 3 Emotion and Development Branch, National Institute of Mental Health, Bethesda, MD USA

**Correction: Research on Child and Adolescent Psychopathology**

10.1007/s10802-025-01362-6

In the original version of this article, the first four sentences under the "Participants" section has been updated from "Data for the present study was collected as part of the [Redacted] study at the [Redacted Institution] (Redacted Institution), a longitudinal cohort study focused on the characterization and treatment of depression in adolescents (data openly available at [details omitted for double-blind peer review]). The cohort encompassed both adolescents with depression and nonpatient controls, monitoring their development and progress over the span of several years. Recruitment began in December of 2018 and is ongoing. Participants were recruited from a metropolitan region and identified through locally distributed postcards aimed at identifying adolescents exhibiting symptoms of MDD." to "Data for the present study was collected as part of the Characterization and Treatment of Depression (CAT-D) study at the National Institute of Mental Health (NIMH), a longitudinal cohort study focused on the characterization and treatment of depression in adolescents (Sadeghi et al., 2022; CAT-D data openly available at https://github.com/transatlantic-comppsych/CATD-study). The cohort encompassed both adolescents with depression and nonpatient controls, monitoring their development and progress over the span of several years. Recruitment began in December of 2018. Participants were recruited primarily from the Washington metropolitan region, identified through locally distributed postcards aimed at identifying adolescents exhibiting symptoms of MDD.". The update has been made due to unblinded data in the affected sentences. The original article has been corrected.

