# Supplementary material for: Temporal Predictions from Anhedonia To Anxiety in Adolescents with Major Depressive Disorder
Source: Res Child Adolesc Psychopathol. 2025 Aug 27;53(11):1595–610. doi: 10.1007/s10802-025-01362-6 (PMC12586218; doi:10.1007/s10802-025-01362-6)
Supplement: Supplementary file 1 — Supplementary Material 1 [file 10802_2025_1362_MOESM1_ESM.docx]

**Supplementary Online Content**

**eFigure 1**

**
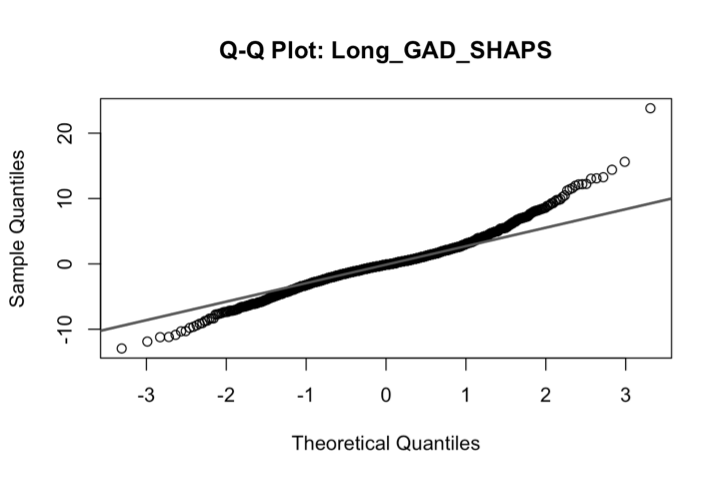

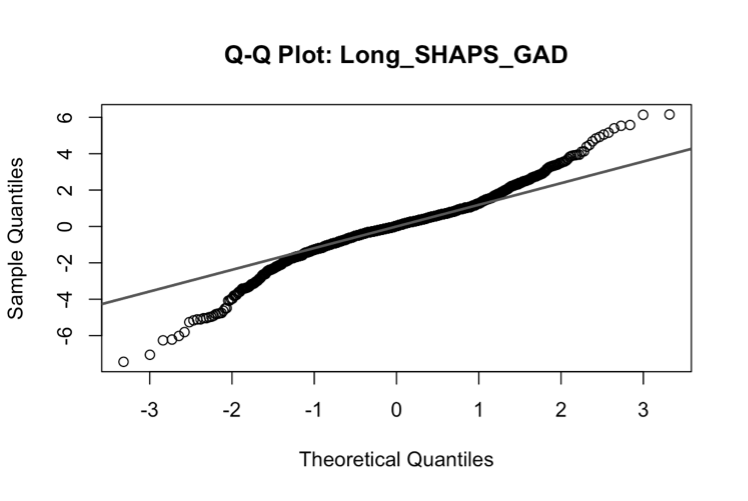

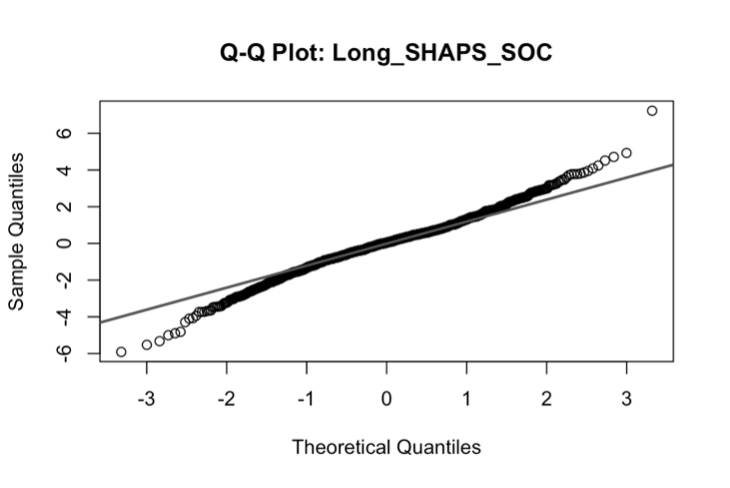

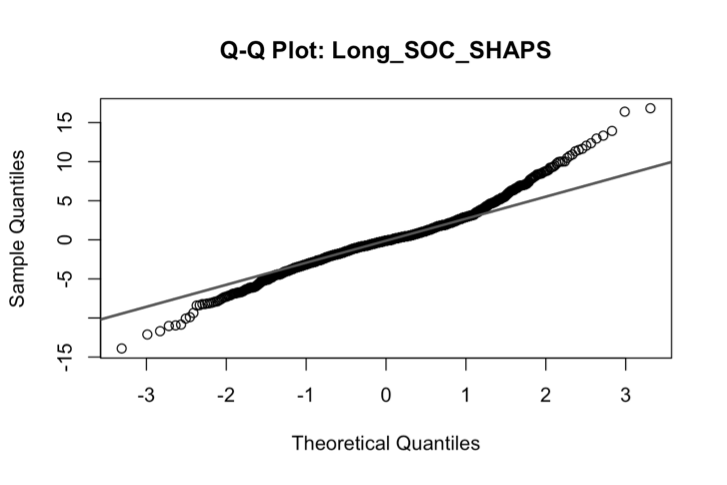

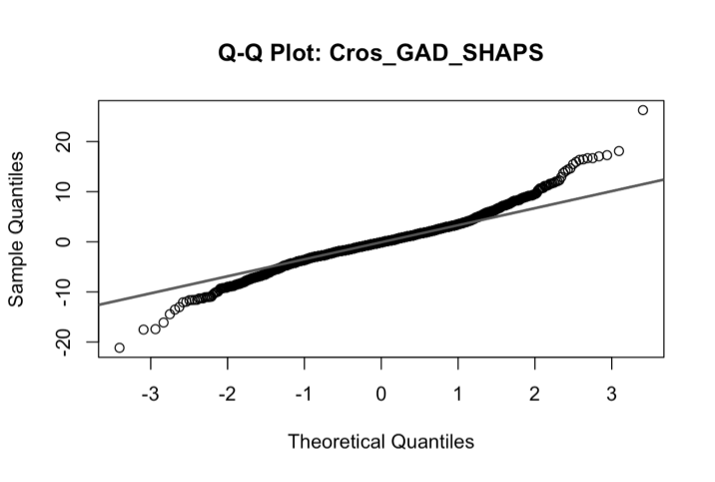

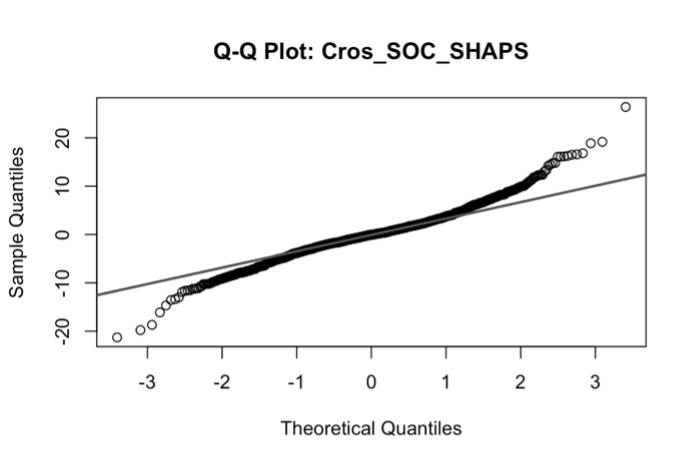
***Q-Q Plots of Level-1 Residuals*

*Note.* SOC = Social anxiety, GAD = Generalized anxiety, SHAPS = Anhedonia. Cros_ = cross-sectional models, Long_ = longitudinal models. For longitudinal models, the first variable listed is the predictor.
